# Allele-specific silencing of EEC p63 mutant R304W restores p63 transcriptional activity

**DOI:** 10.1038/cddis.2016.118

**Published:** 2016-05-19

**Authors:** F Novelli, A M Lena, E Panatta, W Nasser, R Shalom-Feuerstein, E Candi, G Melino

**Affiliations:** 1 Department of Experimental Medicine and Surgery, University of Rome 'Tor Vergata', Rome, Italy; 2 Department of Genetics and Developmental Biology, The Ruth and Bruce Rappaport Faculty of Medicine, Technion-Israel Institute of Technology, Haifa, Israel; 3 Medical Research Council, Toxicology Unit, Leicester University, Hodgkin Building, Leicester, UK

## Abstract

EEC (ectrodactily-ectodermal dysplasia and cleft lip/palate) syndrome is a rare genetic disease, autosomal dominant inherited. It is part of the ectodermal dysplasia disorders caused by heterozygous mutations in *TP63* gene. EEC patients present limb malformations, orofacial clefting, skin and skin's appendages defects, ocular abnormalities. The transcription factor p63, encoded by *TP63*, is a master gene for the commitment of ectodermal-derived tissues, being expressed in the apical ectodermal ridge is critical for vertebrate limb formation and, at a later stage, for skin and skin's appendages development. The ΔNp63*α* isoform is predominantly expressed in epithelial cells and it is indispensable for preserving the self-renewal capacity of adult stem cells and to engage specific epithelial differentiation programs. Small interfering RNA (siRNA) offers a potential therapy approach for EEC patients by selectively silencing the mutant allele. Here, using a systemic screening based on a dual-luciferase reported gene assay, we have successfully identified specific siRNAs for repressing the EEC-causing p63 mutant, R304W. Upon siRNA treatment, we were able to restore ΔNp63-WT allele transcriptional function in induced pluripotent stem cells that were derived from EEC patient biopsy. This study demonstrates that siRNAs approach is promising and, may pave the way for curing/delaying major symptoms, such as cornea degeneration and skin erosions in young EEC patients.

EEC syndrome (ectrodactily-ectodermal dysplasia and cleft lip/palate syndrome, OMIM#604292) is an autosomal dominant rare disorder characterized by abnormal development of tissues that originate from ectoderm. Clinical features include: limbs malformations, orofacial clefting, skin, skin's appendage and ocular defects.^1, 2, 3, 4^ The severity and type of ectodermal defects is highly variable and they may depend on the type of mutations.^5^ EEC patients often present skin manifestation, such as hyperkeratosis, erythroderma, skin erosion and ocular defects because of limbal stem cell deficiency (LSCD).^2, 4^ Limbal stem cells, located in the limbus, at the cornea periphery, are indispensable for cornea formation. EEC patients undergo to a progressive degeneration of the corneal epithelial tissue, generally reaching a peak in the third decades, leading to corneal opacity, poor vision, neovascularization and irreversible blindness.^3, 6, 7, 8^

The transcription factor p63, encoded by *TP63* gene, part of the p53 gene family, is a master gene for the commitment of ectodermal-derived tissues. The action of p63 is likely to be required for skin development and for the formation of the apical ectodermal ridge (AER), which has a critical role in distal outgrowth and patterning of the vertebrate limb and, subsequently, in skin and skin's appendages formation.^6^ Missense mutations in *TP63* are the cause of at least seven rare ectodermal dysplasia (ED) disorders, including EEC.^9, 10^ *TP63* is a complex gene, generating six different proteins because of the use of two alternative promoters (P1 and P2) that give rise to the N-terminal truncated and full-length isoforms, respectively, ΔNp63 and TAp63, and alternative C-terminus splicing, generating *α*, *β* and *γ* isoforms. The ΔNp63α is the predominant isoform expressed in epithelial cells, in different epithelial tissues, including, epidermis, thymic epithelial cells, cornea, prostate, mammary gland, it is indispensable for preserving the self-renewal capacity of adult stem cells.^11, 12, 13, 14^ This is also confirmed by the ΔNp63 isoform-specific knock-out mice phenotype,^15^ which nicely recapitulate human ED symptoms. Besides stemness, p63 is also responsible for regulating different cellular pathways, including cellular metabolism and anti-oxidant response, cellular adhesiveness and cytoskeleton, epithelial differentiation program, cellular migration.^16, 17, 18, 19, 20, 21^

The current therapy for disorders caused by p63 mutations is limited to surgery and is supportive and not curative. Interestingly, five p63 DNA-binding mutations account for almost 90% of EEC patient cases. The five missense mutations affect arginine residues (R204, R227, R279, R280 and R304) and represent hotspot mutations for EEC.^1^ These mutants, similarly to other p63 mutants, are translated into proteins with enhanced stability,^22^ maintain the ability to bind DNA but, being transcriptionally deficient themselves and by inhibiting transactivation by the residual p63 wild-type allele, have a strong dominant-negative effect.^1, 9, 22^ The lack of therapies and the presence of hotspot mutants in EEC patients, lead us to explore the possibility that using allele-specific gene silencing it could selectively inhibit the expression of the disease-associated allele without suppressing the expression of the wild-type allele, therefore restoring the single functional p63 allele, still sufficient for healthy tissue development and maintenance. Here, we designed and selected effective small interfering RNAs (siRNAs) molecules for ΔNp63-R304W EEC mutant using transfected cell line, and examined the efficiency of allele-specific knockdown. We also demonstrated that knockdown of ΔNp63-R304W allele restore ΔNp63-WT allele transcriptional activity. These results indicate that allele-specific silencing of the mutant mRNA can potentially be considered as a therapeutic procedure in EEC, and, more in general, ED patients because of p63 point mutations. This strategy could be relevant to cure/prevent/delay selected clinical features such as skin erosion and loss of corneal function in younger patients.

## Results

### Design and selection of optimal siRNA molecules

**Figure 1:**
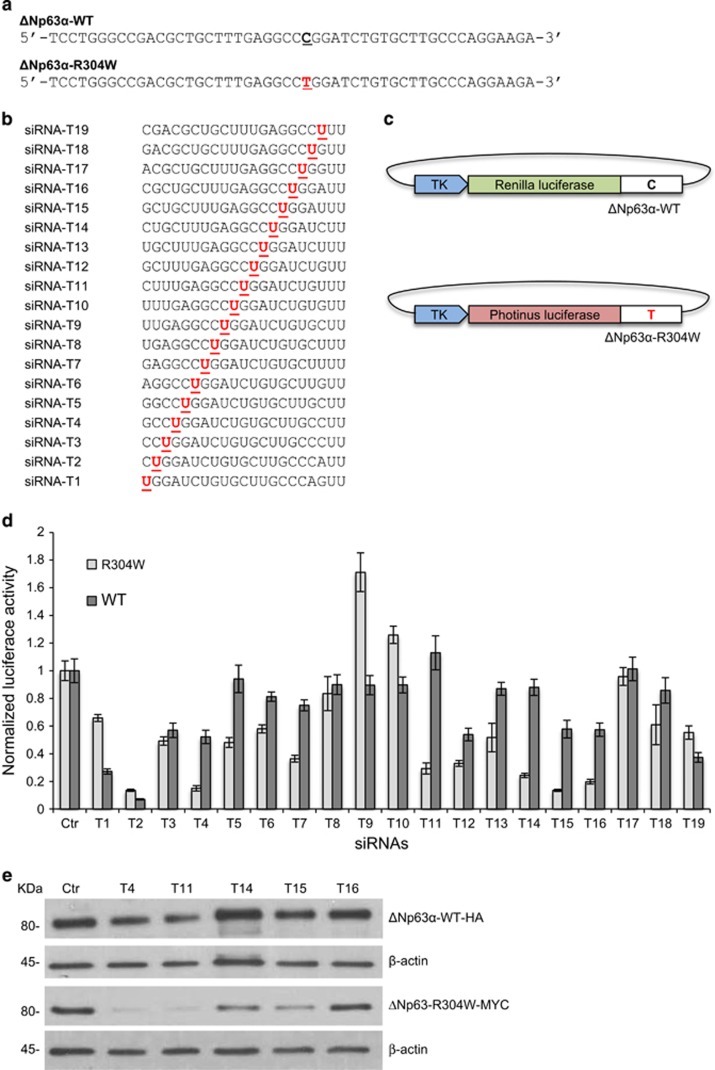
A comprehensive siRNA walk for p63-R304W mutant silencing. (**a**) Nucleotide sequence of WT and mutant p63 transcript. WT and mutated sequences around mutation site (in bold) are shown. (**b**) In order to screen all possible sequences containing the R304W mutation, 19 siRNAs were designed, each of which consisting of 19 nucleotides with two uracil nucleotides overhangs. (**c**) Schematic drawing of reporter alleles. Reporter alleles were constructed based on Photinus and Renilla luciferase reporters driven by TK promoter. WT, Renilla luciferase gene with sequence of WT p63 in 3'-UTR; Mutant, Photinus luciferase gene with sequence of p63-R304W in 3'-UTR. (**d**) Each siRNA under evaluation was co-transfected into HEK-293E cells, at 40 nM, with either a WT-p63 luciferase reporter construct (pRL-TK-WT) and the mutant p63 luciferase reporter construct (pGL-TK-R304W). The sequence walk shows that several siRNAs were selective for the mutant sequence. The best are T4, T11, T14, T15 and T16. Luciferase activity was normalized also for transfection efficiency by *β-*galactosidase activity (PSV-*β*gal, co-transfection) and nonspecific siRNA was used as a negative control (Ctr). Values are calculated relative to nonspecific siRNA using average of three to six experiments. Data are presented as mean±S.D. (**e**) HEK-293E cells were transfected with HA-tagged ΔNp63-WT (pcDNA-HA-ΔNp63) and Myc-tagged ΔNp63-R304W mutant (pcDNA-Myc-ΔNp63-R304W) constructs and five representative discriminatory siRNAs (T4, T11, T14, T15 and T16) at 40 nM. These data show that siRNAs T4 and T11 are highly potent and highly specific for the p63 R304W mutated allele, inhibiting at high-level mutant protein expression but not ΔNp63-WT. One representative experiment of three is shown. *β*-Actin is shown as loading control

A sequence walk was performed whereby all 19 possible siRNA molecules were designed to span and include ΔNp63-R304W point mutation, as shown in Figure 1. For screening of siRNA molecules blocking ΔNp63-R304W expression with little or no effect on ΔNp63-WT, we used two kinds of artificial report constructs^23^ harboring *Renilla* luciferase or *Photinus* luciferase and 50-bp siRNA-targeted sequences with/without C>T substitution in their 3'-UTRs (Figures 1a and c). Each siRNA molecule was investigated for its ability to suppress luciferase activities of ΔNp63-R304W mutated constructs significantly compared with those of ΔNp63-WT ones by transient transfection in HEK-293E epithelial cells.

### Preferential inhibition of ΔNp63-R304W *versus* ΔNp63-WT by specific siRNAs

In order to identify mutated allele-specific siRNAs, we performed luciferase assay upon HEK-293E transient transfection using the reported vector described in Figures 1a and c. Results indicated that almost all siRNAs tested suppressed the luciferase activities of mutated and WT constructs. Some of them were not discriminatory, inhibiting both ΔNp63-WT and ΔNp63-R304W mutant (T1, T2, T8 and T12). Some siRNAs tested had little or no effect on luciferase activity of either ΔNp63-WT or ΔNp63-R304W mutant reporter constructs (T9, T10 and T17). From the 19 siRNAs tested, 10 molecules, T3, T4, T5, T6, T7, T11, T14, T15, T16 and T18, were able to specifically suppress luciferase activity of the mutant allele-containing reporter construct with little effects on the one bearing the WT allele (Figure 1d). Although all of these had good potential for therapeutic use, we further validated them focusing on those suppressing luciferase activity to a maximum of 40% for ΔNp63-WT allele and at least 80% for ΔNp63-R304W mutant allele (T4, T11, T14, T15 and T16). We then analyzed the gene suppression efficiencies of the selected siRNAs on WT/R304W full-length transcripts. HEK-293E cells were transfected with HA-tagged wild-type (pcDNA-HA-ΔNp63) and Myc-tagged mutant (pcDNA-Myc-ΔNp63-R304W) constructs, as an *in vitro* cellular model of the EEC disease-related heterozygous status, and the five representative discriminatory siRNAs (T4, T11, T14, T15 and T16) at a concentration of 40 nM. The data show that siRNAs T4 and T11 are highly potent and highly specific for the p63 mutation R304W at the level of protein expression (Figure 1e), whereas T14, T15 and T16 were less efficient to knockdown ΔNp63-R304W mutant at protein level. This experiment indicates that the siRNAs identified, especially T4 and T11, are selective for the mutated allele, strongly knocking-down the ΔNp63-R304W mutant transcript, resulting in mutated protein translation inhibition.

### Silencing of ΔNp63-R304W mutant partially rescues ΔNp63*α*-WT transcriptional activity

**Figure 2:**
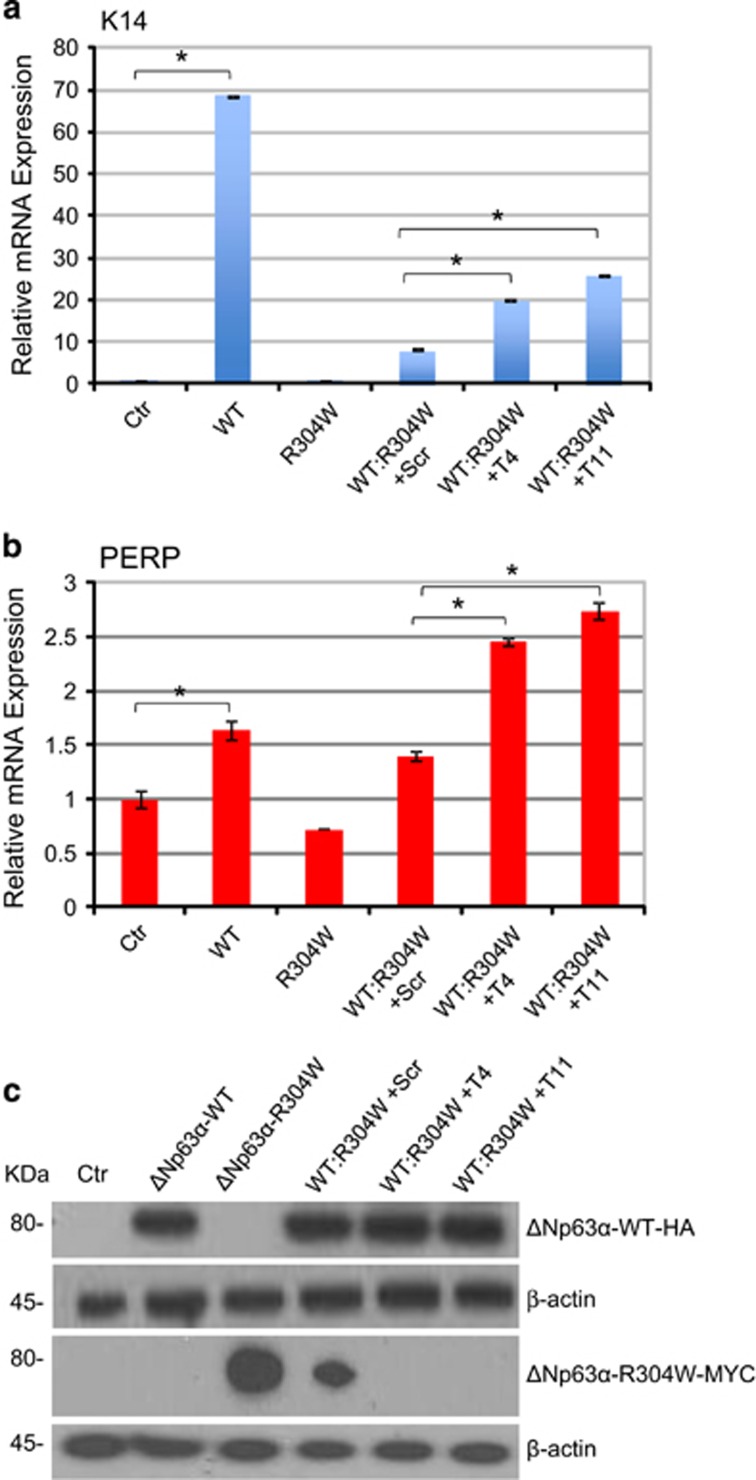
Silencing of the mutant allele restores ΔNp63-WT transcription activity. (**a**) HEK-293E cells were transfected with ΔNp63-WT, ΔNp63-R304W, ΔNp63-WT:ΔNp63-R304W (2:1 ratio) in presence of siRNA not specific sequence (Scr), T4 and T11. ΔNp63-WT is HA-tagged; ΔNp63-R304W is Myc-tagged. T4 and T11 partially restore ΔNp63-WT transcriptional activity, evaluated by real-time PCR of keratin 14 (K14) mRNA. Data are presented as mean±S.D. of three independent experiments. *P<0.05. (**b**) Experiment was performed as described in a) T4 and T11 partially restore ΔNp63-WT transcriptional activity, evaluated by real-time PCR of PERP mRNA. Data are presented as mean±S.D. of three independent experiments. *P<0.05. (**c**) Western blot demonstrates T4 and T11 selectivity. One representative experiment of three is shown. *β*-Actin is shown as loading control

Having demonstrated the effectiveness and the selectivity of the siRNAs identified for ΔNp63-R304W mutant allele, we checked whether these (T4 and T11) could restore the transcriptional activity of ΔNp63-WT. As experimental system, we used HEK-293E cells monitoring the endogenous ΔNp63-driven targets keratin 14 (K14) and p53 effector related to PMP-22 (PERP) mRNAs expression. HEK-293E cells were transiently transfected with ΔNp63-WT, ΔNp63-R304W and ΔNp63-WT:ΔNp63-R304W (2:1 ratio) in presence of not specific siRNA (Scr), T4 or T11. K14 mRNA increases 68-fold after ΔNp63-WT transient transfection, whereas its transcriptional activity is strongly inhibited by the presence of ΔNp63-R304W that has a well-known dominant-negative effect on ΔNp63-WT transcriptional activity, also at 2:1 ratio (Figure 2a). Interestingly, addition of 40 nM T4 or T11 partially rescued ΔNp63-WT function, increasing K14 mRNA to 2.2- and 2.8-fold, respectively, in comparison with ΔNp63-WT:ΔNp63-R304W transfected cells with not specific siRNA (Scr) (Figure 2a). Similar results were obtained for PERP mRNA. Indeed, addition of 40 nM T4 or T11 partially rescued ΔNp63-WT function, increasing PERP mRNA to 1.8- and 2.2-fold, respectively, in comparison with ΔNp63-WT:ΔNp63-R304W transfected cells with not specific siRNA (Scr) (Figure 2b). Western blot confirmed T4 and T11 efficacy of selective silencing (Figure 2c). These results were also confirmed in Saos-2 cell line. ΔNp63-WT, ΔNp63-R304W and ΔNp63-WT:ΔNp63-R304W (2:1 ratio) in presence of not specific siRNA (Scr), T4 or T11, were transiently transfected in Saos-2 cells and obtained partial or total rescue of K14, PERP and EVPL mRNAs (Supplementary Figures S1a and b), because of allele selective silencing by T4 and T11. These results show that selective silencing of the ΔNp63-R304W mutated allele restore ΔNp63-WT transcriptional activity, as shown by the significant increases in endogenous p63 targets mRNAs (K14, PERP and EVPL) upon siRNAs treatments.

### Rescue of corneal epithelial differentiation of EEC-iPSCs by mutated-p63 allele-specific siRNAs

**Figure 3:**
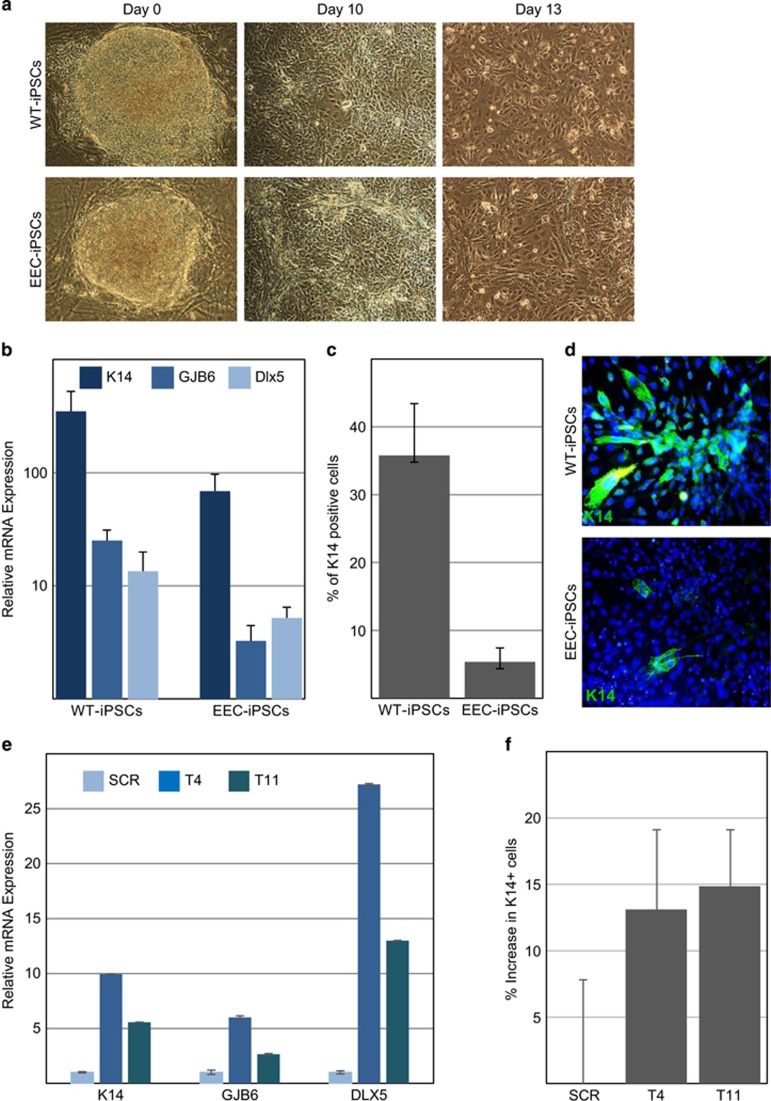
Rescue of corneal epithelial differentiation of EEC-iPSCs by mutated-p63 allele-specific silencing RNAs. WT-iPSCs and EEC-iPSCs were subjected to corneal epithelial differentiation protocol. Phase contrast pictures of WT-iPSCs and EEC-iPSCs at days 0, 10 and 13 of differentiation are shown in **a**. Cells were harvested at days 13–15 of differentiation and subjected to RT-qPCR analysis of the indicated genes (**b**), flow cytometry analysis (**c**) and immunofluorescence staining (**d**) of K14. EEC-iPSCs were transfected with nonspecific control silencing RNA (SCR) or specific siRNAs against p63 mutated allele (T4 and T11) at days 8–10 during differentiation into corneal epithelial cells. Cells were harvested at day 13–15 and subjected to RT-qPCR analysis of the indicated genes (**e**) and flow cytometry analysis of K14 (**f**). Gene expression was normalized to GAPDH housekeeping gene (**b** and **e**) and data represent the fold change in gene expression compared with undifferentiated cells (**b**) or compared with SCR (**e**). Flow cytometry data represent the percentage of K14+ cells in the cell population (**c**) and the relative increase in the K14+ cells compared with SCR (**f**). Results are presented as mean±S.D. (Scale bar, 100 *μ*m)

Based on the efficient repression of p63 mutant targeting siRNAs in HEK-293E cells, we aimed at testing the effect in a more physiologically relevant model using patients' cells. We have recently generated induced pluripotent stem cells (iPSCs) from p63-R304W-EEC patients and showed that these cells can serve as an *in vitro* model for testing novel therapeutic approaches.^7^ In contrast to primary cultures of patient keratinocyte, iPSCs provide a durable model because they can be expanded forever in an undifferentiated state and directed to lineage-specific fates including epidermal and corneal lineages, whereas EEC patient cells have a very short life span. To test the possibility that siRNA can restore patient-specific iPSCs (EEC-iPSCs), we chose to study corneal differentiation because of the high efficiency of this differentiation model.^24^ Normal iPSCs (WT-iPSCs) and EEC-iPSCs were seeded on collagen IV-coated dishes in the presence of medium conditioned by human corneal fibroblasts. As illustrated in Figures 3a and b, iPSC lines underwent sequential differentiation into corneal fate and displayed epithelial characteristics as evident by their morphology and gene expression. As shown in Figures 3b and d, although WT-iPSC could fully commit into epithelial lineage, EEC-iPSC displayed partial differentiation as evident by reduced expression of K14, and two other additional p63-target genes, GJ6B and Dlx5.^7^ Accordingly, we used this system to test whether mutated allele-specific siRNA can restore the function of p63 mutant function and promote corneal differentiation of EEC-iPSC. For that purpose, EEC-iPSCs were subjected to differentiation and transfection with specific siRNAs (T4 and T11) or control siRNA (Scr) was performed at days 8–10 of differentiation because at this stage cells begin to express p63 and undergo commitment from ectoderm to stratified epithelium.^24^ Cells were collected at days 13–15 of differentiation and the impact of siRNA treatment on cell differentiation was determined by real-time qPCR and flow cytometry analysis of K14. Interestingly, under transfection of T4 or T11, cell differentiation was partially rescued (Figures 3e and f). GJ6B, which is linked with ED and, previously shown, to be elevated in WT mice but not in p63-null mice at embryonic day 14.5,^6^ was rescued by T4 and T11 (six and threefold, respectively). Similarly, the mRNA level of DLX5, a p63-target gene that is associated with EEC syndrome, was enhanced by T4 and T11, 27- and 13-fold, respectively. Finally, real-time PCR and flow cytometry analyses confirmed silencing efficacy of T4 and T11 with an increase in K14 mRNA (9- and 5.5-folds, respectively; Figure 3e) and in the fraction of K14-positive cells (Figure 3f).

## Discussion

In this study, we developed a method for allele-specific gene silencing with siRNAs that target a *TP63* R304W missense mutation, found in patients affected with autosomal dominant EEC syndrome. EEC syndrome, part of ED disorders, is caused by p63 mutations localized in the DNA-binding domain (DBD). Several efforts to understand the mechanism of disease in EEC have shown that p63 DBD mutants have both loss their ability to transactivate target genes and also have increased protein stability,^22^ resulting in a strong dominant-negative effect toward the p63-WT allele.^1, 22^ As *TP63* encodes an essential protein, indispensable to maintain adult stem cells proliferative potential and to engage specific differentiation programs, treatment would necessitate to selectively inhibit the mutant allele expression without affecting WT allele. Several methods to modulate gene expression have been developed, including gene editing by zinc-finger nuclease (TALEN, CRISPR/Cas) and modification of the transcripts by exon skipping, and protein inhibitors by antibodies and drugs.^25, 26, 27^ Among these methods, we believe that allele-specific knockdown could be a highly relevant therapeutic method for EEC syndrome, and possibly other ED disorders, because the mutated-p63 protein form a high order complex with WT p63 protein (tetramerization), therefore showing the dominant-negative effect.

**Figure 4:**
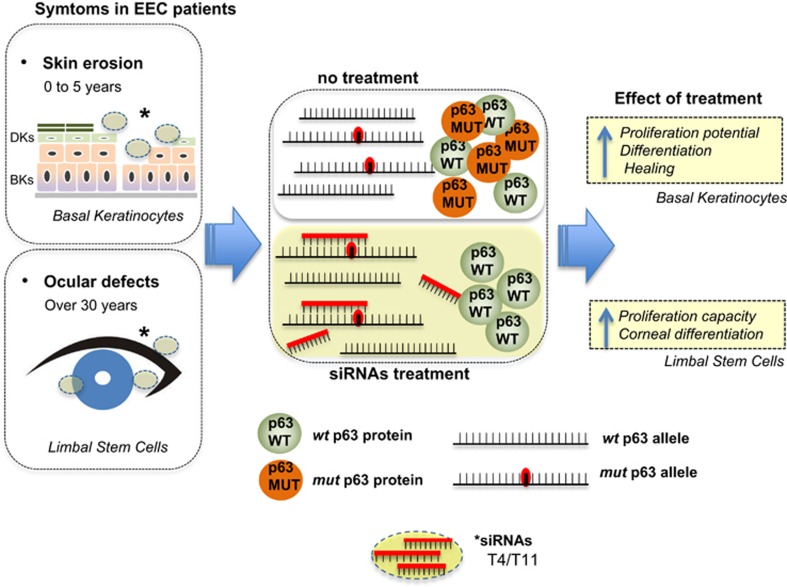
Schematic representation of siRNA-based therapeutic strategy in EEC disease. siRNAs identified could be used topically to cure/delay selected clinical onset, with high impact in the patient quality of life, such as skin erosion and ocular defects. Treatment with selected siRNAs (T4/T11 for p63-R304W) will silence the expression of p63 mutated allele, allowing p63-WT to transcriptionally activate target genes and to rescue proliferation and differentiation in basal keratinocytes (BK) and limbal stem cells (LSC)

Five hotspot missense mutations, accounting for 90% of the mutations identified in EEC, have been identified, among them R304W, suggesting that by selecting few siRNA molecules one would be able to treat the majority of the EEC patients.^1^ We think that the siRNAs identified could be used topically to cure/delay selected clinical onsets with high impact on the patient quality of life, by attenuating skin erosion and ocular defects (Figure 4). EEC patients often have skin manifestation, some patients experience aplasia cutis congenital of the scalp and back, a debilitation clinical manifestation with no treatment. In these patients, skin defects heal very slowly and may recur periodically throughout childhood. In addition, EEC patients result in a specific ocular phenotype-involving cornea. The cornea epithelium is regenerated physiologically by p63-expressing limbal stem cells. The major case of ocular defect in these patients is LSCD, resulting in a progressive degeneration of corneal epithelial tissue, generally reaching a peak on the third and fourth decade of patients' life. When this occurs, conjuctival epithelium migrates over the cornea leading to cornea opacifiation, neovascularization, poor vision and irreversible blindness. Such slow aggravation in corneal abnormalities provides a large window for preventive intervention. Our results suggest that preventive strategies applied in early stage of the disease, could help young patients to correct/delay the EEC defects. Depending on the severity of the symptoms, siRNA molecules could be applied topically, as body cream or eye drops formulations, following specific indications (Figure 4). Similarly, a preventive strategy may be applied to counteract dermatological defects in EEC patients. The use of siRNAs in dermatological dominant-negative disorders have been explored also in other studies for keratin mutations in epidermolysis bullosa simplex^28^ and epidermolitic palmoplantar keratoderma.^29^ In ocular disease, the use of allele-specific siRNA silencing has been investigated in Meesmann epithelial corneal dystrophy^30, 31, 32^ and in Lattice corneal dystrophy type I.^33^ Altogether, this strategy could be widely applied for treating dominantly inherited pathologies and is currently under investigation in clinical trials.

Here, we demonstrated a proof-of-principle approach to select highly potent and mutant-specific siRNA molecules that target p63 mutated allele causing EEC syndrome. The next challenge is the development of efficacious non-invasive delivery system to get these small molecules to the ocular surface and to cross skin outer layers. A similar strategy could be used also for other p63-dependent ED pathologies.

## Materials and Methods

### Cell culture and cells transfection

HEK-293E cells were cultured in Dulbecco's modified Eagle medium (DMEM-F12, Lonza, Basel, Switzerland) supplemented with 10% fetal bovine serum (Gibco, Life Technologies, Carlsbad, CA, USA). For transfection, HEK-293E cells were seeded at 1 × 10^6^ cells into 10 cm Petri dishes. Cells were transfected using Lipofectamine 2000 (Invitrogen, Carlsbad, CA, USA) according to the manufacturer's instructions. HEK-293E cells were transfected with HA-tagged ΔNp63-WT (pcDNA-HA-ΔNp63*α*) and Myc-tagged ΔNp63-R304W mutant (pcDNA-Myc-ΔNp63*α*-R304W) constructs and R304W mutation-specific siRNAs T4 and T11 at a concentration of 40 nM. The total amount of transfected DNA was 14 *μ*g per dish. A nonspecific control siRNA was used as negative control (Ctrl). Where required, empty pcDNA-HA vector was added to ensure equal total amounts of plasmid DNA in each transfection.

### Design and screening of siRNAs

Complementary DNA sequences consisting of a 50-bp fragment of ΔNp63-WT and R304W mutant were cloned into pRL-TK (*Renilla luciferase*) and pGL-TK (*Photinus luciferase*) vectors, respectively. Site-directed mutagenesis primers were designed manually based on p63 sequence information. In order to screen all possible sequences containing the R304W mutation, 19 siRNAs were designed, each of which consisting of a 19-nucleotide sequence with two uracil nucleotide overhangs. Each siRNA molecule under evaluation was co-transfected into HEK-293E cells, seeded at 100 000 cells per well in a 12-well plate, at a concentration of 40 nM with either a wild-type p63 luciferase reporter construct (pRL-TK-WT) or a mutant p63 luciferase reporter construct (pGL-TK-R304W). Luciferase activity was measured using the luciferase reporter assay 48 h after transfection. PSV-*β*gal reporter vector was also co-transfected and *β*-galactosidase activity was used for transfection efficiency normalization. Not specific siRNA was used as a negative control.

### Western blot analysis

HEK-293E cells were incubated for 48 h after transfection, then washed with PBS and lysed with Trypsin 1x. The extracted protein samples were denatured at 98 °C for 10 min before being resolved on a SDS-10% polyacrylamide gels at 10 *μ*g concentration. Gels were then transferred using polyvinylidene fluoride membranes. The expression of HA-tagged and Myc-tagged constructs was detected with an anti-HA monoclonal mouse antibody (Abcam, Cambridge, UK, 1:500 dilution) and an anti-Myc monoclonal mouse antibody (Celle Signalling Technology, Danvers, MA, USA, 1:500 dilution), respectively. An anti-*β*-actin antibody (Sigma, Saint Louis, MO, USA, 1:50000 dilution) was used as an endogenous control. Membranes were washed with PBS-Tween for 10 min three times before being stained with anti-mouse secondary antibody (Sigma,1 : 10 000 dilution) for 1 h at room temperature. Detection was performed with ECL kit (Perkin Elmer, Waltham, MA, USA).

### Real-time qPCR

Total RNA was extracted by the RNAeasy kit (Qiagen, Hilden, Germany) and quantified by spectrophotometric analysis. Total RNA (500ng) was used for reverse transcription using GoScript Reverse Transcription System kit (Promega, Fritchburg, WI, USA) following the manufacturer's instructions. Human *β*-actin mRNA was used as housekeeping gene for quantity normalization. Primer pairs used in PCR reactions are:

*β*-act forward 5′-CTGGCACCACACCTTCTACAATG-3′, *β*-act reverse 5′-TAGCACAGCCTGGATAGCAAC-3′. K14 forward 5′-CGACCTGGAAGTGAAGATCCG-3′, K14 reverse 5′-CACACTCATGCGCAGGTTCAA-3′. PERP forward 5′-TGTGGTGGAAATGCTCCCAAG-3′, reverse 5′-CCAATCACTCTCAGGAAGACAAGC-3′. EVPL forward 5′-TGCCCGAGCACAACATCCTG-3′, reverse 5′-GACGCCGCCTTCAGTAGGTCTC-3′.

### iPSC differentiation and transfection

Human corneal fibroblasts were isolated from cadaveric cornea as described previously^24^ and grown in DMEM (Invitrogen) 10% FCS. Cell growth was arrested by mitomycin treatment (8 *μ*g/ml; Sigma) for 3 h followed by washing with medium and incubation overnight. For the preparation of conditioned medium, growth medium was replaced by epithelial medium containing 60% DMEM+GlutMax, 30% F12, 10% fetal clone II, 5 *μ*g/ml insulin, 0.5 *μ*g/ml hydrocortisone, 10 ng/ml EGF, 0.2 mM adenine and 10 mM choleratoxin. Conditioned media were collected (20 ml per T75 flask) every day for up to 10 days and stored at −20 ºC.

iPSC were grown and differentiated as described previously.^7, 24^ In order to induce the differentiation of the iPSC lines into corneal epithelial cells, iPSC lines were seeded on collagen IV-coated dishes (0.5 mg/ml; Sigma) in the presence of conditioned media. iPSCs were transfected using Lipofectamine RNAiMAX (Invitrogen) according to the manufacturer's instructions with nonspecific control siRNA (SCR) or mutated allele-specific siRNAs (T4, T11), on days 8–10 during differentiation at a concentration of 40 nM and medium was replaced the next day.

### Analysis of iPSC differentiation

Cells were harvested after 13–15 days of differentiation and subjected to the assays below. RNA was extracted using TRI Reagent (Sigma) and cDNA was synthesized from 2 *μ*g RNA using MultiScribe MuLV Reverse Transcription Kit (Applied Biosystems, Foster City, CA, USA). qRT-PCR was performed in triplicates using SYBR Green (Bioline, London, UK) and specific primers (sequences available on request). Each reaction contained 15 *μ*l primers and SYBR-Green PCR Master Mix and 5 *μ*l cDNA. Human GAPDH mRNA was used as housekeeping gene for normalization. The relative expression of each transcript was calculated as a fold change relative to normalized control sample of differentiated iPSC transfected with SCR. Immunofluorescent staining of K14 and imaging as well as flow cytometry was performed as previously reported.^7, 24^ Briefly, cells were fixed with acetone for 10 min at −20 ºC. After washing with PBS, cells were incubated with 0.5% BSA, 0.5% saponin and donkey serum for 30 min. Primary antibody for K14 (Millipore, Billerica, MA, USA) was added for 45 min at room temperature. Acquisition was performed on FACS-CyAn using CellQuest software (BD Biosciences, Franklin Lakes, NJ, USA).

## Abbreviations

EEC: ectrodactily-ectodermal dysplasia and cleft lip/palate
ED: ectodermal dysplasia
AER: apical ectodermal ridge
LSCD: limbal stem cell deficiency
siRNA: small interfering RNA
DBD: DNA-binding domain

## Supplementary Material

Suppelementary Information

## References

[bib1] Rinne T, Brunner HG, van Bokhoven H. p63-associated disorders. Cell Cycle 2007; 6: 262–268. 17224651 10.4161/cc.6.3.3796

[bib2] Di Iorio E, Kaye SB, Ponzin D, Barbaro V, Ferrari S, Böhm E et al. Limbal stem cell deficiency and ocular phenotype in ectrodactyly-ectodermal dysplasia-clefting syndrome caused by p63 mutations. Ophthalmology 2012; 119: 74–83. 21959367 10.1016/j.ophtha.2011.06.044

[bib3] Clements SE, Techanukul T, Coman D, Mellerio JE, McGrath JA. Molecular basis of EEC (ectrodactyly, ectodermal dysplasia, clefting) syndrome: five new mutations in the DNA-binding domain of the TP63 gene and genotype-phenotype correlation. Br J Dermatol 2010; 162: 201–207. 19903181 10.1111/j.1365-2133.2009.09496.x

[bib4] Celik TH, Buyukcam A, Simsek-Kiper PO, Utine GE, Ersoy-Evans S, Korkmaz A et al. A newborn with overlapping features of AEC and EEC syndromes. Am J Med Genet 2011; 155A: 3100–3103. 22065614 10.1002/ajmg.a.34328

[bib5] Rinne T, Hamel B, van Bokhoven H, Brunner HG. Pattern of p63 mutations and their phenotypes—update. Am J Med Genet 2006; 140: 1396–1406. 16691622 10.1002/ajmg.a.31271

[bib6] Shalom-Feuerstein R, Lena AM, Zhou H, De La Forest Divonne S, Van Bokhoven H, Candi E et al. ΔNp63 is an ectodermal gatekeeper of epidermal morphogenesis. Cell Death Differ 2011; 18: 887–896. 21127502 10.1038/cdd.2010.159 PMC3131930

[bib7] Shalom-Feuerstein R, Serror L, Aberdam E, Müller FJ, van Bokhoven H, Wiman KG et al. Impaired epithelial differentiation of induced pluripotent stem cells from ectodermal dysplasia-related patients is rescued by the small compound APR-246/PRIMA-1MET. Proc Natl Acad Sci USA 2013; 110: 2152–2156. 23355677 10.1073/pnas.1201753109 PMC3568301

[bib8] Felipe AF, Abazari A, Hammersmith KM, Rapuano CJ, Nagra PK, Peiro BM. Corneal changes in ectrodactyly-ectodermal dysplasia-cleft lip and palate syndrome: case series and literature review. Int Ophthalmol 2012; 32: 475–480. 22618129 10.1007/s10792-012-9585-6

[bib9] Celli J, Duijf P, Hamel BC, Bamshad M, Kramer B, Smits AP et al. Heterozygous germline mutations in the p53 homolog p63 are the cause of EEC syndrome. Cell 1999; 99: 143–153. 10535733 10.1016/s0092-8674(00)81646-3

[bib10] van Bokhoven H, Hamel BC, Bamshad M, Sangiorgi E, Gurrieri F, Duijf PH et al. p63 Gene mutations in eec syndrome, limb-mammary syndrome, and isolated split hand-split foot malformation suggest a genotype-phenotype correlation. Am J Hum Genet 2001; 69: 481–492. 11462173 10.1086/323123 PMC1235479

[bib11] Rivetti di Val, Cervo P, Lena AM, Nicoloso M, Rossi S, Mancini M et al. p63-microRNA feedback in keratinocyte senescence. Proc Natl Acad Sci USA 2012; 109: 1133–1138. 22228303 10.1073/pnas.1112257109 PMC3268329

[bib12] Candi E, Cipollone R, Rivetti di Val Cervo P, Gonfloni S, Melino G, Knight R. p63 in epithelial development. Cell Mol Life Sci 2008; 65: 3126–3133. 18560758 10.1007/s00018-008-8119-x PMC11131713

[bib13] Candi E, Rufini A, Terrinoni A, Giamboi-Miraglia A, Lena AM, Mantovani R et al. DeltaNp63 regulates thymic development through enhanced expression of FgfR2 and Jag2. Proc Natl Acad Sci USA 2007; 104: 11999–12004. 17626181 10.1073/pnas.0703458104 PMC1924561

[bib14] Viganò MA, Lamartine J, Testoni B, Merico D, Alotto D, Castagnoli C et al. New p63 targets in keratinocytes identified by a genome-wide approach. EMBO J 2006; 25: 5105–5116. 17036050 10.1038/sj.emboj.7601375 PMC1630419

[bib15] Romano RA, Smalley K, Magraw C, Serna VA, Kurita T, Raghavan S et al. ΔNp63 knockout mice reveal its indispensable role as a master regulator of epithelial development and differentiation. Development 2012; 139: 772–782. 22274697 10.1242/dev.071191 PMC3265062

[bib16] Latina A, Viticchiè G, Lena AM, Piro MC, Annicchiarico-Petruzzelli M, Melino G et al. ΔNp63 targets cytoglobin to inhibit oxidative stress-induced apoptosis in keratinocytes and lung cancer. Oncogene 2015; (doi:10.1038/onc.2015.222; e-pub ahead of print). 10.1038/onc.2015.222 26096935

[bib17] Viticchiè G, Agostini M, Lena AM, Mancini M, Zhou H, Zolla L et al. p63 supports aerobic respiration through hexokinase II. Proc Natl Acad Sci USA 2015; 112: 11577–11582. 26324887 10.1073/pnas.1508871112 PMC4577137

[bib18] Carroll DK, Carroll JS, Leong CO, Cheng F, Brown M, Mills AA et al. p63 regulates an adhesion programme and cell survival in epithelial cells. Nat Cell Biol 2006; 8: 551–561. 16715076 10.1038/ncb1420

[bib19] Candi E, Rufini A, Terrinoni A, Dinsdale D, Ranalli M, Paradisi A et al. Differential roles of p63 isoforms in epidermal development: selective genetic complementation in p63 null mice. Cell Death Differ 2006; 13: 1037–1047. 16601749 10.1038/sj.cdd.4401926

[bib20] Rufini A, Weil M, McKeon F, Barlattani A, Melino G, Candi E. p63 protein is essential for the embryonic development of vibrissae and teeth. Biochem Biophys Res Commun 2006; 340: 737–741. 16410075 10.1016/j.bbrc.2005.12.065

[bib21] Ihrie RA, Marques MR, Nguyen BT, Horner JS, Papazoglu C, Bronson RT et al. Perp is a p63-regulated gene essential for epithelial integrity. Cell 2005; 120: 843–856. 15797384 10.1016/j.cell.2005.01.008

[bib22] Browne G, Cipollone R, Lena AM, Serra V, Zhou H, van Bokhoven H et al. Differential altered stability and transcriptional activity of ΔNp63 mutants in distinct ectodermal dysplasias. J Cell Sci 2011; 124: 2200–2207. 21652629 10.1242/jcs.079327

[bib23] Noguchi S, Ogawa M, Kawahara G, Malicdan MC, Nishino I. Allele-specific gene silencing of mutant mRNA restores cellular function in ullrich congenital muscular dystrophy fibroblasts. Mol Ther Nucleic Acids 2014; 3: e171. 24959844 10.1038/mtna.2014.22 PMC4078762

[bib24] Shalom-Feuerstein R, Serror L, De La Forest Divonne S, Petit I, Aberdam E, Camargo L et al. Pluripotent stem cell model reveals essential roles for miR-450b-5p and miR-184 in embryonic corneal lineage specification. Stem Cells 2012; 30: 898–909. 22367714 10.1002/stem.1068

[bib25] Melo SP, Lisowski L, Bashkirova E, Zhen HH, Chu K, Keene DR et al. Somatic correction of junctional epidermolysis bullosa by a highly recombinogenic AAV variant. Mol Ther 2014; 22: 725–733. 24390279 10.1038/mt.2013.290 PMC3982486

[bib26] Keswani SG, Balaji S, Le L, Leung A, Lim FY, Habli M et al. Pseudotyped adeno-associated viral vector tropism and transduction efficiencies in murine wound healing. Wound Repair Regen 2012; 20: 592–600. 22713157 10.1111/j.1524-475X.2012.00810.x PMC3654388

[bib27] Sebastiano V, Zhen HH, Haddad B, Bashkirova E, Melo SP, Wang P et al. Human COL7A1-corrected induced pluripotent stem cells for the treatment of recessive dystrophic epidermolysis bullosa. Sci Transl Med 2014; 6: 264ra163. 10.1126/scitranslmed.3009540 PMC4428910 25429056

[bib28] Atkinson SD, McGilligan VE, Liao H, Szeverenyi I, Smith FJ, Moore CB et al. Development of allele-specific therapeutic siRNA for keratin 5 mutations in epidermolysis bullosa simplex. J Invest Dermatol 2011; 131: 2079–2086. 21716320 10.1038/jid.2011.169

[bib29] Leslie Pedrioli DM, Fu DJ, Gonzalez-Gonzalez E, Contag CH, Kaspar RL, Smith FJ et al. Generic and personalized RNAi-based therapeutics for a dominant-negative epidermal fragility disorder. J Invest Dermatol 2012; 132: 1627–1635. 22402445 10.1038/jid.2012.28

[bib30] Liao H, Irvine AD, Macewen CJ, Weed KH, Porter L, Corden LD et al. Development of allele-specific therapeutic siRNA in Meesmann epithelial corneal dystrophy. PLoS One 2011; 6: e28582. 22174841 10.1371/journal.pone.0028582 PMC3236202

[bib31] Allen EH, Atkinson SD, Liao H, Moore JE, Leslie Pedrioli DM et al. Allele-specific siRNA silencing for the common keratin 12 founder mutation in Meesmann epithelial corneal dystrophy. Invest Ophthalmol Vis Sci 2013; 54: 494–502. 23233254 10.1167/iovs.12-10528 PMC3869971

[bib32] Courtney DG, Atkinson SD, Allen EH, Moore JE, Walsh CP, Pedrioli DM et al. siRNA silencing of the mutant keratin 12 allele in corneal limbal epithelial cells grown from patients with Meesmann's epithelial corneal dystrophy. Invest. Ophthalmol Vis Sci 2014a; 55: 3352–3360. 10.1167/iovs.13-12957 24801514

[bib33] Courtney DG, Atkinson SD, Moore JE, Maurizi E, Serafini C, Pellegrini G et al. Development of allele-specific gene-silencing siRNAs for TGFBI Arg124Cys in lattice corneal dystrophy type I. Invest Ophthalmol Vis Sci 2014b; 55: 977–985. 24425855 10.1167/iovs.13-13279

