# Supplementary material for: Allele-specific silencing of EEC p63 mutant R304W restores p63 transcriptional activity
Source: Cell Death Dis. 2016 May 19;7(5):e2227–. doi: 10.1038/cddis.2016.118 (PMC4917656; doi:10.1038/cddis.2016.118)
Supplement: Suppelementary Information [file cddis2016118x1.pdf]

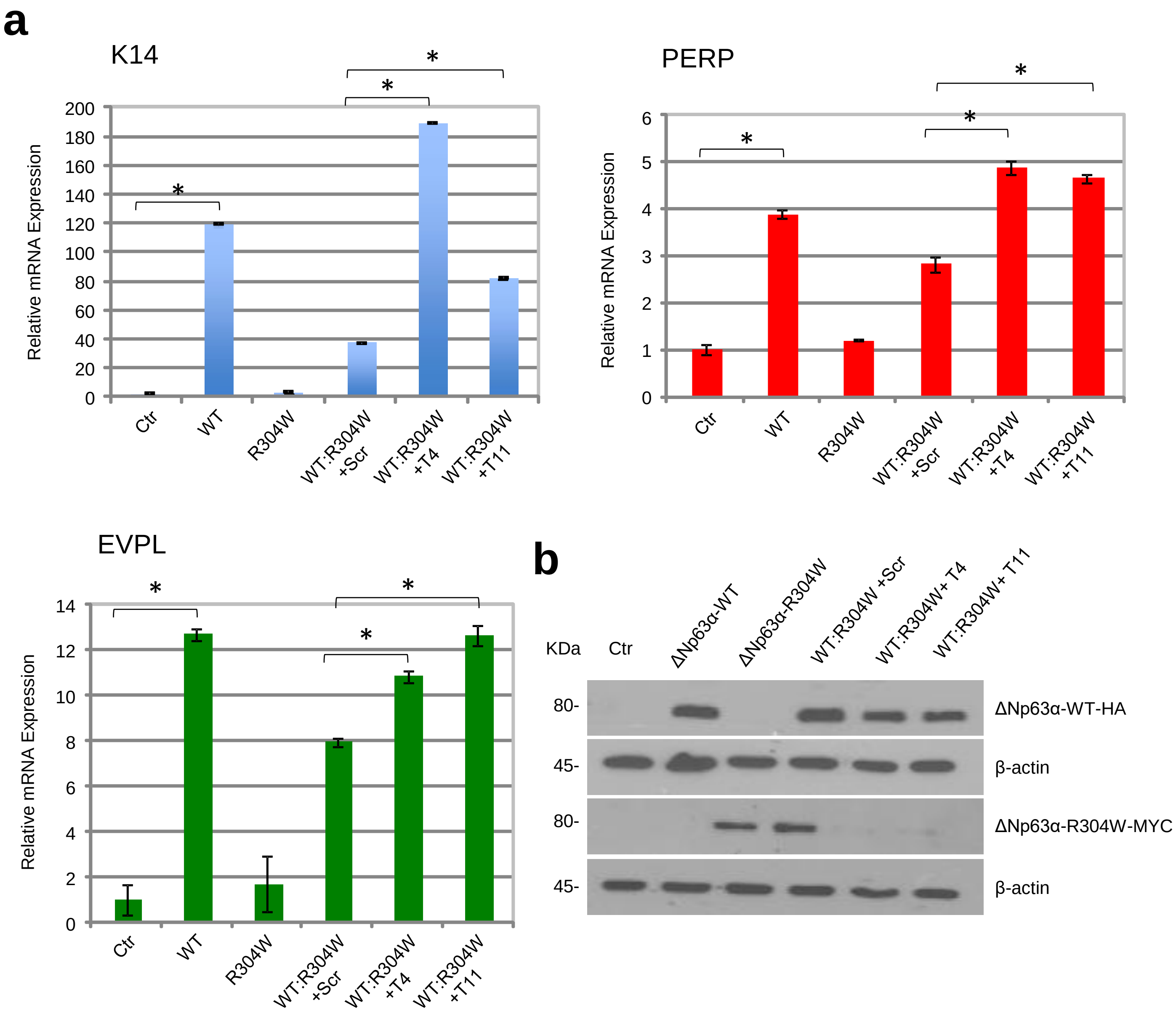

Figure S1 Silencing of the mutant allele restores DNp63-WT transcription activity in Saos-2 cells. Saos-2 cells were transfected with DNp63-WT, DNp63-R304W, DNp63-WT:DNp63-R304W (2:1 ratio) in presence of siRNA not specific sequence (Scr), T4 and T11. ΔNp63-WT is HA-tagged; ΔNp63-R304W is Myc-tagged. *, $p<0.05$. **a)** T4 and T11 partially restore DNp63-WT transcriptional activity, evaluated by real-time PCR of keratin 14 (K14), PERP and EVPL mRNAs. Data are presented as mean ± SD of three independent experiments. *, $p<0.05$. **b)** Western blot demonstrates the T4 and T11 selectivity. One representative experiment of three is shown. b-actin is shown as loading control.
